# Peculiar morphology of Asgard archaeal cells close to the prokaryote-eukaryote boundary

**DOI:** 10.1128/mbio.00327-25

**Published:** 2025-04-16

**Authors:** Burak Avcı, Kassiani Panagiotou, Mads Albertsen, Thijs J. G. Ettema, Andreas Schramm, Kasper Urup Kjeldsen

**Affiliations:** 1 Section for Microbiology, Department of Biology, Aarhus University 1006, Aarhus, Denmark; 2 Laboratory of Microbiology, Wageningen University and Research 568404, Wageningen, Gelderland, The Netherlands; 3 Center for Microbial Communities, Aalborg University 1004, Aalborg, Denmark; 4 Center for Electromicrobiology, Department of Biology, Aarhus University 1006, Aarhus, Denmark; University of California, Irvine, California, USA

**Keywords:** eukaryogenesis, Asgard archaea, Hodarchaeales, marine sediments, *in situ *hybridization, fluorescence microscopy, cellular complexity

## Abstract

The emergence of complex eukaryotic cells from prokaryotic ancestors is a major enigma in the history of life. Current data suggest that Asgard archaea are the closest prokaryotic relatives of eukaryotes and have a genetic potential for cellular complexity, suggesting their key role in the evolution of eukaryotes. The Asgard archaeal order Hodarchaeales was recently proposed as the sister lineage of eukaryotes with a unique set of eukaryotic signature proteins. However, there is no microscopic evidence to show the cellular structure of these closest known archaeal relatives of eukaryotes. Here, we retrieved Hodarchaeales-affiliated full-length 16S rRNA sequences from marine sediments (Aarhus Bay, Denmark), representing 0.1% of the relative rRNA read abundance in sequence libraries, and designed new oligonucleotide probes specifically targeting their cells. We then employed catalyzed reporter deposition-fluorescence *in situ* hybridization (CARD-FISH) and imaged the labeled cells by super-resolution microscopy with appropriate controls. Hodarchaeales-affiliated cells were characterized by an elongated cell body connected to a rounded expansion at one pole with a confined central DNA localization. They were conspicuously large, with an average length of 3 µm, ranging from 1.5 to 5.2 µm. The average width was 1.1–0.8 µm in the round expansion and the cell body, respectively. The remarkable size and morphology of the detected Hodarchaeales-related cells suggest a potential for complex eukaryote-like cellular architecture in these as-yet-uncultivated Asgard archaea, which could represent a key transitional stage in eukaryogenesis.

**IMPORTANCE:**

Asgard archaea played a pivotal role in the evolution of complex cellular life, as recent data revealed their close relationship to eukaryotes in the Tree of Life and a genetic repertoire suggesting intricate cellular organization. This suggests that the key elements of eukaryotic cellular complexity originated in Asgard archaea. However, visual evidence to show their cellular structure and morphological diversity remains scarce, leaving an open question of how the genetic potential for cellular complexity translates into phenotype. In this study, we report the remarkable size and unusual shape of yet-uncultivated Asgard archaeal cells, which are close to the prokaryote-eukaryote boundary. Our findings suggest a previously unknown complex cellular structure in the closest archaeal relatives of eukaryotes and could contribute to our understanding of the evolution of complex life forms.

## OBSERVATION

Asgard archaea comprise the closest prokaryotic relatives of eukaryotes (1–5), supporting the hypothesis that eukaryotes likely evolved from an ancient endosymbiosis between an Asgard archaeal host cell (6) and an alphaproteobacteria-related symbiont (7). Also, Asgard archaeal genomes are enriched in genes encoding eukaryotic signature proteins (ESPs) that form the hallmarks of eukaryotic cells (1–4). This suggests that some of the essential components of eukaryotic cellular complexity already evolved before the emergence of the first eukaryote.

Microscopic examinations of two closely related members of the Asgard archaeal order Lokiarchaeales revealed complex surface structures characterized by long and often branching membrane protrusions (8, 9). Cryo-electron tomography further revealed the presence of a dynamic actin cytoskeleton consisting of twisted, double-stranded Lokiactin filaments, which extend throughout the membrane protrusions and cell bodies, serving as a scaffold for an elaborate cellular architecture (9). However, eukaryote-like compartmentalization was not observed. On the other hand, we previously demonstrated spatial separation of DNA and ribosomes in distantly related Lokiarchaeales and Heimdallarchaeales cells, suggesting a potential for sub-cellular compartmentalization in some environmental Asgard archaea (10). Therefore, Asgard archaeal cell structures appear to be diverse but remain poorly characterized.

By comprehensive phylogenomic analyses, a recent study resolved the branching point of the eukaryotes within Asgard archaea, identifying Hodarchaeales as the closest known extant prokaryotic relatives of the eukaryotes (4). In agreement, Hodarchaeales genomes are enriched in ESPs involved in the cytoskeleton and ubiquitin system and encode a unique set of ESPs associated with information processing (4). However, the morphology of Hodarchaeal cells is unknown, and whether the genetic potential for cellular complexity translates into phenotype remains an open question.

**Fig 1:**
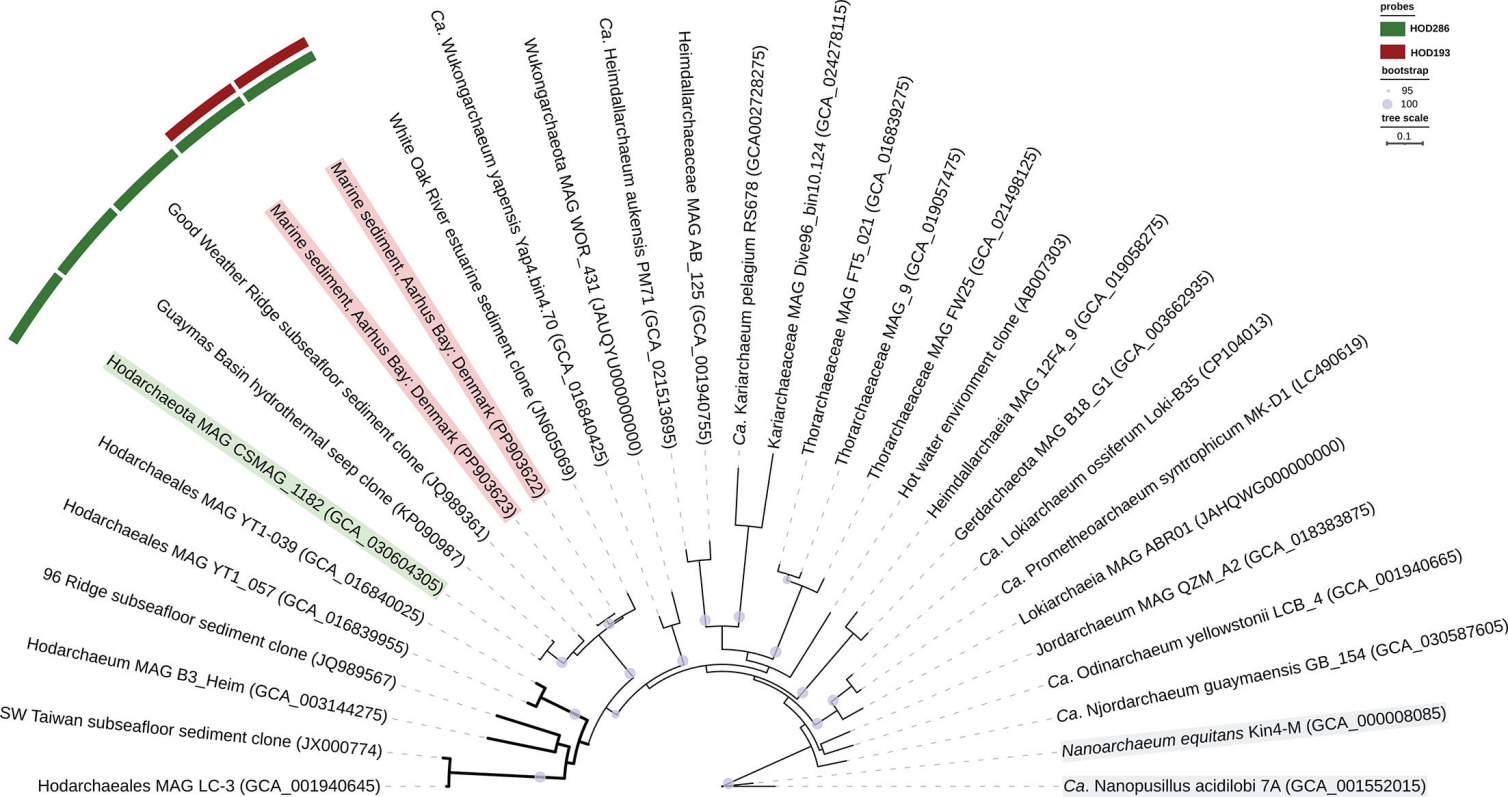
Phylogenetic analysis of Hodarchaeales-related 16S rRNA sequences retrieved from Aarhus Bay sediments. Maximum likelihood phylogeny of two Hodarchaeales-affiliated operational taxonomic units (red) and related Asgard archaeal 16S rRNA sequences is shown. Specificities of FISH probes are highlighted in green and red. 16S rRNA sequence of the metagenome-assembled genome CSMAG_1182 is marked with green. Hodarchaeales sequences are depicted with the bold tree branch. Nanoarchaeota sequences (gray) are used as the outgroup. Lavender circles indicate nodes receiving >95% bootstrap support (100 replications). The scale bar shows 10% nucleotide sequence divergence.

We retrieved full-length Hodarchaeales-affiliated 16S rRNA sequences from Aarhus Bay sediments, representing two operational taxonomic units (OTUs), with a relative abundance of 0.1% in 16S rRNA sequence libraries from 5 to 10 and 15 to 20 cm sediment depths. These sequences share 88%–93% sequence similarity with Hodarchaeal 16S rRNA sequences previously sampled from estuarine and marine sediments (Fig. 1). They also cluster with the 16S rRNA gene sequence of the metagenome-assembled genome (MAG) CSMAG_1182 (11) with ~90% sequence similarity and form a sister clade to the other Hodarchaeales MAGs. Although the phylogenetic reconstruction confidently placed these OTUs branching as a sister group to Hodarchaeales 16S rRNA sequences, we could not resolve the phylogenomic position of CSMAG_1182 due to its low quality, 54% completeness, 67% strain heterogeneity, and encoding only about one-third of the archaeal marker genes assessed from the Genome Taxonomy Database (GTDB) (12). Despite a comprehensive screening of the Asgard archaeal genome diversity available in the National Center for Biotechnology Information (NCBI) database as of 28 June 2024, we could not identify another MAG that is phylogenetically related to CSMAG_1182 or contains a 16S rRNA gene sequence closely related to these two OTUs. Therefore, the recovery of relevant Hodarchaeales genomes from environmental samples will be necessary to accurately infer the phylogenomic position of the retrieved Hodarchaeales-affiliated OTUs.

We designed two oligonucleotide probes, HOD193 and HOD286, both specifically targeting the two Hodarchaeales-affiliated 16S rRNA sequence OTUs from Aarhus Bay sediments without any match outside the target group in the SILVA database (v.132) (Fig. 1; Table S1). These probes facilitated the dual labeling of targeted Hodarchaeales-related cells with two distinct fluorescent dyes, ensuring confident identification of true-positive signals in heterogeneous sediment samples.

**Fig 2:**
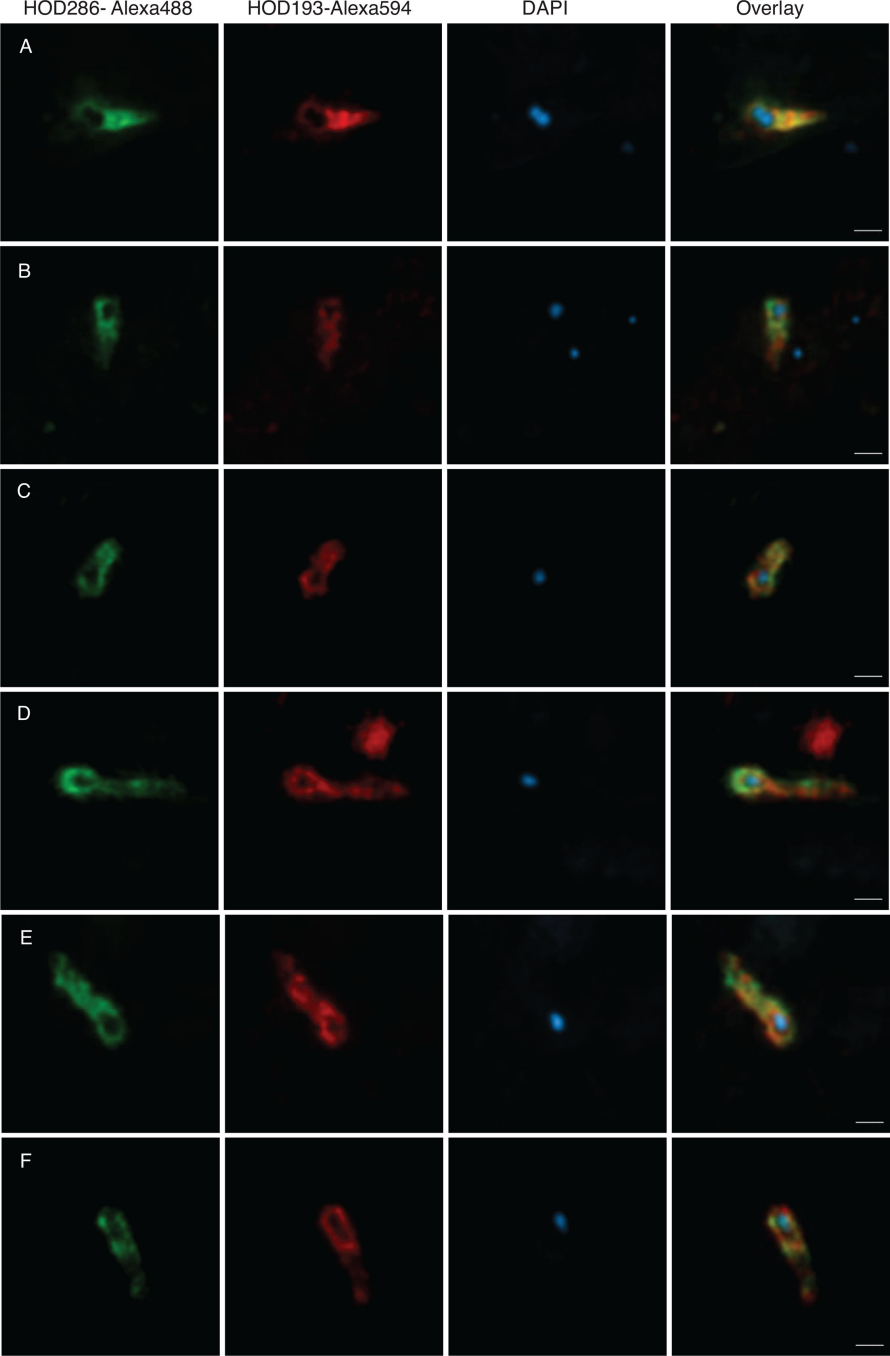
Visualization of Hodarchaeales-related cells in Aarhus Bay sediments using CARD-FISH. Probe names and dyes are depicted for each panel. Three representative images for the morphotypes with short (3–1.5 µm, **A–C**) and long (3–5 µm, **D–F**) cell lengths are shown. The cells were imaged in a super-resolution confocal laser scanning microscope. Maximum intensity projection of the acquired z-stack images is depicted. Z-stack images used in maximum intensity projections are shown in Fig. S1. Control experiments are included in Fig. S4. Images represent dual-labeled Hodarchaeales-related cells (*n* = 40) in four individual experiments. The scale bar is 1 µm.

We imaged CARD-FISH labeled Hodarchaeales-affiliated cells by super-resolution confocal laser scanning microscopy. The detected cells featured a long cell body with a rounded expansion at one pole (Fig. 2; Fig. S1). 4′,6-Diamidino-2-phenylindole (DAPI) staining revealed condensed DNA signals only in a confined area at the center of the expanded cell pole (Fig. 2), which were spatially separated from ribosome-derived CARD-FISH signals with limited overlaps but lacking a conspicuous gap between the two signals (Fig. S2). Such signal patterns were previously detected in Lokiarchaeales and Heimdallarchaeales cells with spherical central DAPI signals, suggesting a potential for compartmentalization (10). Nucleoid formation with condensed DNA was also previously described, for example, in the Thaumarchaeota *Cenarcheum symbiosum* (13) and *Nitrosopumilus maritimus* (14), indicating diverse DNA organizations in archaea. Furthermore, the detected Hodarchaeales-affiliated cells lack membrane protrusions like those observed in cultivated Lokiarchaeales strains (8, 9); however, such cellular structures may not be detectable in our CARD-FISH visualizations due to their presumably lower ribosome content (10).

The average length of Hodarchaeales-affiliated cells was 3,028  ±  864 nm with a wide size range from 1,506 to 5,209 nm as depicted by different morphotypes (*n* = 40) (Fig. 2A through C and D through F). The average widths of the rounded expansion and cell body were 1,094 ± 194 nm and 843 ± 171 nm, respectively (*n* = 40) (Fig. S3). This cellular size, shape, and DNA organization appear clearly distinct from typical benthic bacteria and archaea (15). To the best of our knowledge, this presents one of the largest archaeal cells after *Haloquadratum walsbyi* (up to 12 µm) (16) and *Staphylothermus marinus* (up to 15 µm) (17), both of which exhibit size variability depending on their substrate availability. The large cell size is reminiscent of the smallest known eukaryotic protozoa capable of phagocytosis (18). Importantly, control experiments combining the newly designed probes with non-binding, non-sense probes as well as with the domain-specific eukaryote probe EUK1195 (Table S1), which covers 87% of eukaryotic 18S rRNA sequences in the SILVA database (v. 138.2), proved the absence of non-specific and eukaryotic probe binding and thus confirmed the specific hybridization to Hodarchaeales-related cells (Fig. S4).

Some Hodarchaeales genomes encode putative homologs of MreB and ARP2/3 proteins, which are known to be involved in cell shape determination in bacteria (19) and organize actin filaments into branched networks in eukaryotes (20) to drive membrane-involved cellular processes such as endocytosis and phagocytosis (21). This genetic repertoire may not only explain the distinctive shape of the detected Hodarchaeales-related cells but, together with their remarkable size, might also make phagocytosis conceivable. Notably, phagocytosis could have been a potential mode of interaction of ancestral Asgard archaea with the bacterial partner that later became the mitochondrion (5).

Our comprehensive experimental approach with appropriate controls enabled the reliable detection of extremely rare Hodarchaeales-affiliated cells in Aarhus Bay sediments. The peculiar morphology of detected cells could indicate a hitherto unknown complex cellular organization at the prokaryote-eukaryote interface, aligning with the previous findings on yet-uncultivated Asgard archaeal cells (10). Future studies should aim at better taxon sampling of Hodarchaeales genomes in environmental samples to resolve the phylogenetic diversity within the order and visualize other Hodarchaeales clades. Also, methodological advances in cultivating Asgard archaea from marine sediments and non-invasive labeling of their cells for electron microscopy are required to enable targeted cellular ultrastructure analyses. This will shed further light on the evolution of complex cellular structures and provide essential insights into the origins of eukaryotic life.

## Data Availability

The 16S rRNA sequences are deposited in NCBI’s GenBank with the accession numbers PP903622 and PP903623.

## SUPPLEMENTAL MATERIAL

Supplemental Information mbio.00327-25-s0001.pdf Supplemental materials and methods, figures, and table.

ASM does not own the copyrights to Supplemental Material that may be linked to, or accessed through, an article. The authors have granted ASM a non-exclusive, world-wide license to publish the Supplemental Material files. Please contact the corresponding author directly for reuse.
