# Supplementary material for: Peculiar morphology of Asgard archaeal cells close to the prokaryote-eukaryote boundary
Source: mBio. 2025 Apr 16;16(5):e00327-25. doi: 10.1128/mbio.00327-25 (PMC12077186; doi:10.1128/mbio.00327-25)
Supplement: Supplemental Information — Supplemental materials and methods, figures, and table. [file mbio.00327-25-s0001.pdf]

Supplementary Information

Peculiar Morphology of Asgard Archaeal Cells Close the Prokaryote-Eukaryote Boundary

Burak Avcı[1,2*], Kassiani Panagiotou[2], Mads Albertsen[3], Thijs J. G. Ettema[2], Andreas Schramm[1,4], Kasper Urup Kjeldsen[1*]

[1] Section for Microbiology, Department of Biology, Aarhus University, Aarhus, Denmark.

[2] Laboratory of Microbiology, Wageningen University and Research, Wageningen, The Netherlands.

[3] Center for Microbial Communities, Aalborg University, Aalborg, Denmark

[4] Centre for Electromicrobiology, Department of Biology, Aarhus University, Aarhus, Denmark.

[*]Corresponding authors:

Burak Avcı, Section for Microbiology, Department of Biology, Aarhus University, Ny Munkegade 116, 8000 Aarhus C, Denmark, E-mail: bavci@bio.au.dk

Kasper Urup Kjeldsen, Section for Microbiology, Department of Biology, Aarhus University, Ny Munkegade 116, 8000 Aarhus C, Denmark, E-mail: kasperuk@bio.au.dk

List of contents

1. Materials and methods
2. Supplementary figures and tables

Materials and Methods

Sampling and RNA extraction

Sediment cores were taken by a Rumohr Lot Corer [1] during cruises with the RV Aurora at Aarhus Bay (Denmark) station M5 (56.103333 N, 10.457833 E; water depth 27 m) in October 2023. Subsamples for RNA extraction and catalyzed reporter-deposition-fluorescence *in situ* hybridization (CARD-FISH) were collected as described previously [2]. Briefly, subsamples for RNA extraction were obtained from the sediment surface to 40 cm depth in 5 cm increments and were snap-frozen in liquid nitrogen. For CARD-FISH, muddy sediment samples from 15-20 cm depth were fixed in 1% formaldehyde at room temperature for 1 h. RNA was extracted from partially thawed sediment aliquots using the RNeasy Power Soil total RNA kit (Qiagen, Hilden, Germany) according to the manufacturer's protocol.

Sequencing, phylogenetic analysis, and probe design

Primer-free sequencing of 16S rRNA from Aarhus Bay sediment RNA extracts was performed as described previously [2, 3]. Within this dataset, we identified two Hodarchaeales-affiliated operational taxonomic units (OTUs, 98% sequence identity threshold). We found the closest relatives of these OTUs by blastn search against the NCBI core nucleotide database [4] and by retrieving sequences from the Genome Taxonomy Database [5] that were either classified as Hodarchaeales or shared >88% sequence identity with the two OTUs from Aarhus Bay sediments. All sequences were added to the alignment of the SILVA SSURef NR 99 (v.138) database [6] using the SILVA incremental aligner [7] with subsequent manual curation. The phylogenetic affiliation of the two OTUs was inferred by RAxML (version 7.2.8) [8] maximum likelihood analysis using the GTRGAMMAI model of evolution and the rapid bootstrap

algorithm (100 bootstrap repetitions) together with almost full-length Hodarchaeales-related sequences (>1400 bp) and representative Asgard archaeal sequences from other phyla. The analysis was based on 1213 sequence alignment positions, which were selected by applying a 50% positional conservation filter. The resulting phylogenetic tree was visualized in iTOL (v.6) (Fig. 1) [9].

Oligonucleotide probes were designed using the probe design tool implemented in ARB [10]. Non-target hits at 0,1,2,3 mismatches were checked in SILVA SSURef NR 99 v.138 [6] and NCBI core nucleotide database (blastn search with percent identity >85%, e value <2, and query cover >85) [4] (Table S1). In addition to almost full-length sequences (>1400 bp) indicated in Fig. 1, the probe-target group also includes partial sequences (400-1000 bp) in the NCBI core nucleotide database that shows >95% sequence similarity with two Hodarchaeales-affiliated OTUs retrieved from Aarhus Bay sediments. Hybridization conditions for these probes were optimized as described previously [2]. The highest possible formamide concentration providing dual-labeled signals (HOD193 and HOD286) was used for further hybridizations. As negative control experiments, we combined the newly designed probes with non-sense probes NON-HOD193 and NON-HOD286 (Table S1) to confirm that detected Hodarchaeales-affiliated signals were not random probe-binding or auto-fluorescent particles (Fig. S4). Also, we used the domain-level eukaryote-specific probe EUK1195 (Table S1) to confirm that the observed FISH signals did not originate from the eukaryotic cells (Fig. S4).

Catalyzed reporter deposition in situ *hybridization (CARD-FISH)*

CARD-FISH analyses were performed as described previously [2] with minor modifications. Briefly, the muddy sediment samples were placed on ice, and cells were detached from sediment particles using an HD2070 ultrasonication probe with 10 pulses of 30% intensity given every third second for 30 sec in total (Bandelin, Berlin, Germany). After the gravitational settlement of sediment particles for 30 min on ice, the resulting supernatant was deposited onto a 0.2 µm pore size polycarbonate membrane filter (Millipore, Cork, Ireland). To prevent cell loss, filter sections were embedded in low-gelling temperature agarose. Endogenous peroxidases were inactivated with 0.15% H_2O_2 in methanol (Sigma-Aldrich, St. Louis, MO, USA), and cell wall permeabilization was done with a lysozyme solution (10 mg ml^{-1}) (Sigma-Aldrich, St. Louis, MO, USA) at 37°C for 60 minutes. Whole-cell hybridization was performed with horseradish peroxidase (HRP)-labeled probes (0.5 ng DNA $µl^{-1}$) together with helper probes at 46 °C (Biomers, Ulm, Germany). After washing at 48 °C, signals were amplified using Alexa488-labeled tyramides (1 µg ml^{-1}) (Thermo Fisher, Waltham, MA, USA). HRP from the first probe was inactivated by 3% H_2O_2 solution. Afterwards, hybridization with the second probe was done, and CARD signal-amplification was performed with Alexa594-labeled tyramides (1 µg ml^{-1}) (Thermo Fisher, Waltham, MA, USA). For DNA staining, 4′,6-diamidino-2-phenylindole (DAPI, 1 µg ml^{-1}) (Thermo Fisher, Waltham, MA, USA) was mounted in Citifluor (Electron Microscopy Sciences, Hatfield, PA, USA): Vecta Shield (Vector Laboratories, Burlingame, CA, USA) (4:1 v:v) medium. In total, we analyzed 40 dual probe-labeled Hodarchaeales-affiliated cells in four individual experiments using two samples from a sediment core taken in October 2023 (Fig. 2. Fig. S1, Fig. S2).

Microscopy

Imaging was performed on a Zeiss (Oberkochen, Germany) LSM800 confocal laser scanning microscope (CLSM) with an Airyscan super-resolution detector. 405, 488, and 561 nm lasers and Plan-Apochromat ×63/1.4 oil DIC M27 objective were used. Thin z-sections (0.13 μm) of high-resolution images were collected in 3 rotations for each channel. Airyscan super-resolution reconstruction and maximum intensity projection of the images were done in in ZEN Black software using the recommended settings (Fig. 2, Fig. S1). Cell lengths and widths were determined based on a line intensity profile drawn along the vertical and horizontal axes of the FISH signals (Fig. S3).

References

1. Meischner DR, Rumohr J. A light-weight, high-momentum gravity corer for subaqueous sediments. *Senckenb. Marit*. 1974;**6**:105-17

2. Avcı B, Brandt J, Nachmias D *et al.* Spatial separation of ribosomes and DNA in Asgard archaeal cells. *ISME J*. 2022;**16**:606-10 https://doi.org/10.1038/s41396-021-01098-3

3. Karst SM, Dueholm MS, McIlroy SJ *et al.* Retrieval of a million high-quality, full-length microbial 16S and 18S rRNA gene sequences without primer bias. *Nat. Biotechnol*. 2018;**36**:190-95 https://doi.org/10.1038/nbt.4045

4. Sayers EW, Bolton EE, Brister JR *et al.* Database resources of the national center for biotechnology information. *Nucleic Acids Res*. 2021;**50**:D20-D26 https://doi.org/10.1093/nar/gkab1112

5. Chaumeil P-A, Mussig AJ, Hugenholtz P *et al.* GTDB-tk v2: memory friendly classification with the Genome Taxonomy Database. *Bioinformatics*. 2022;**38**:5315-16 https://doi.org/10.1093/bioinformatics/btac672

6. Quast C, Pruesse E, Yilmaz P *et al.* The SILVA ribosomal RNA gene database project: improved data processing and web-based tools. *Nucleic Acids Res*. 2012;**41**:D590-D96 https://doi.org/10.1093/nar/gks1219

7. Pruesse E, Peplies J, Glöckner FO. SINA: accurate high-throughput multiple sequence alignment of ribosomal RNA genes. *Bioinformatics*. 2012;**28**:1823-29 https://doi.org/10.1093/bioinformatics/bts252

8. Stamatakis A. RAxML version 8: a tool for phylogenetic analysis and post-analysis of large phylogenies. *Bioinformatics*. 2014;**30**:1312-13 https://doi.org/10.1093/bioinformatics/btu033

9. Letunic I, Bork P. Interactive Tree of Life (iTOL) v6: recent updates to the phylogenetic tree display and annotation tool. *Nucleic Acids Res*. 2024;**52**:W78-W82 https://doi.org/10.1093/nar/gkae268

10. Ludwig W, Strunk O, Westram R *et al.* ARB: A software environment for sequence data. *Nucleic Acids Res*. 2004;**32**:1363-71 https://doi.org/10.1093/nar/gkh293

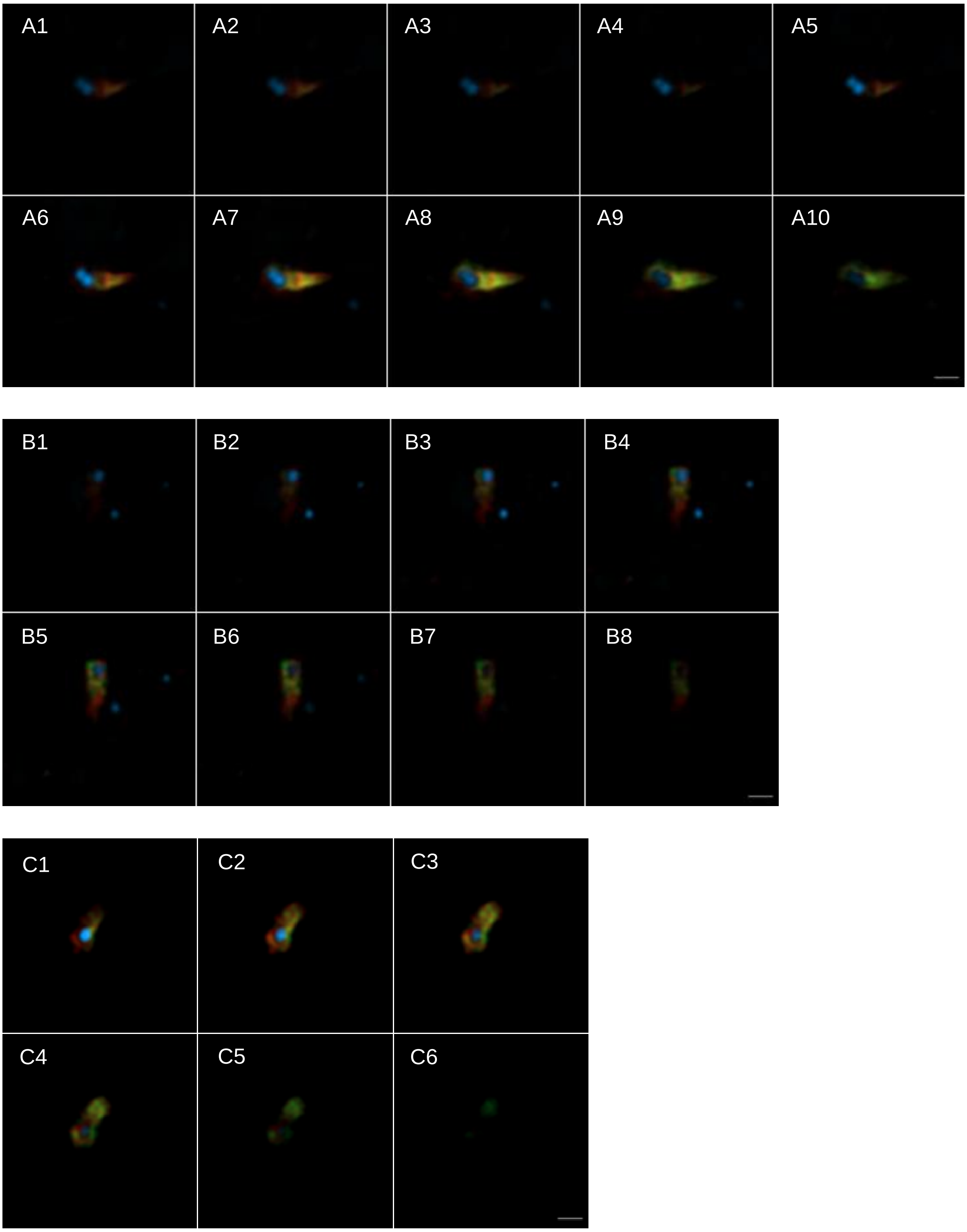
A1
A2
A3
A4
A5
A6
A7
A8
A9
A10
B1
B2
B3
B4
B5
B6
B7
B8
C1
C2
C3
C4
C5
C6

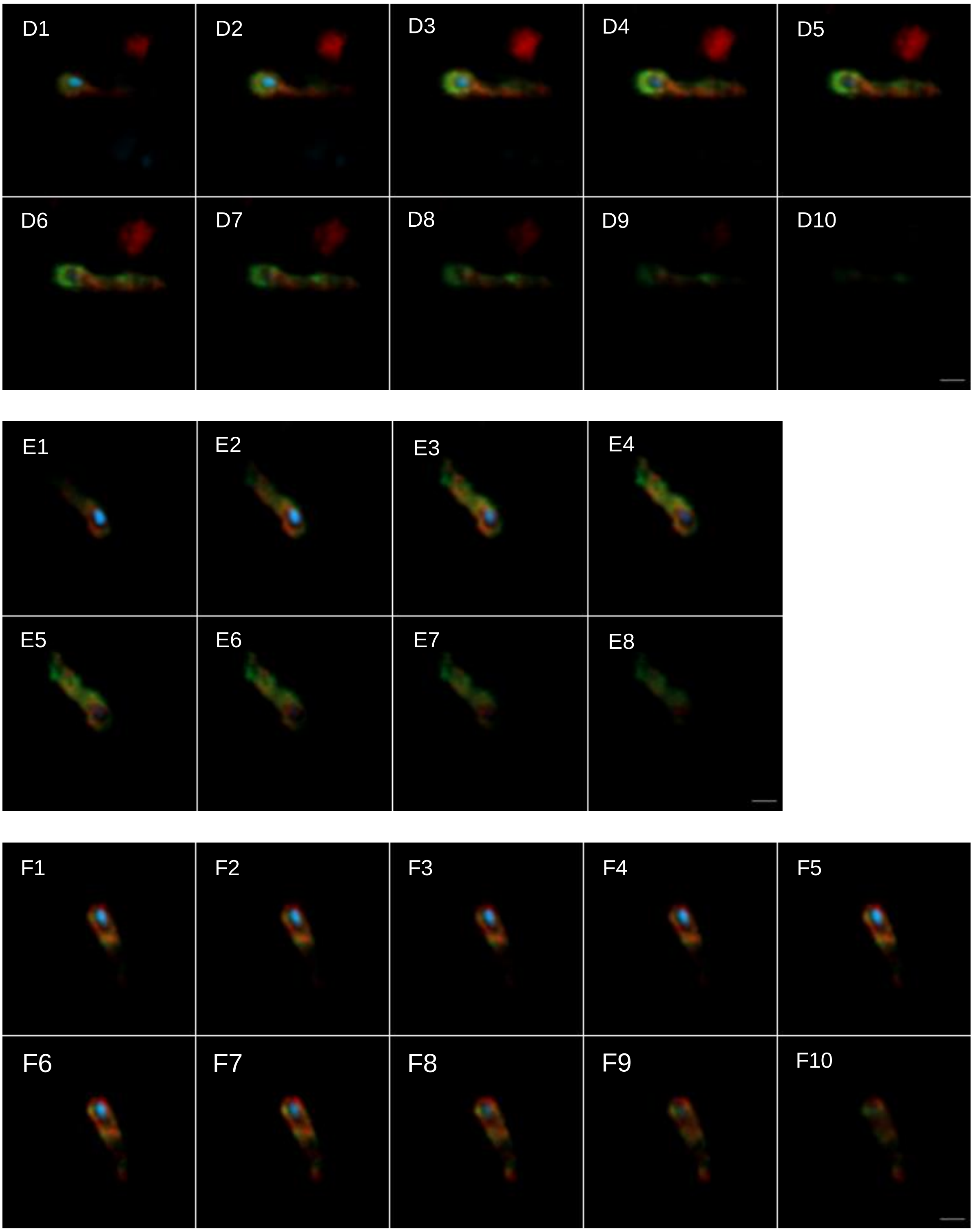

Supplementary Fig. 1. Z-stack images that were used to construct maximum intensity projection of Hodarchaeales-affiliated cells in Figure 2. Overlay of HOD286 (Alexa488), HOD193 (Alexa594), and DAPI signals are depicted. The scale bar is 1 μm.

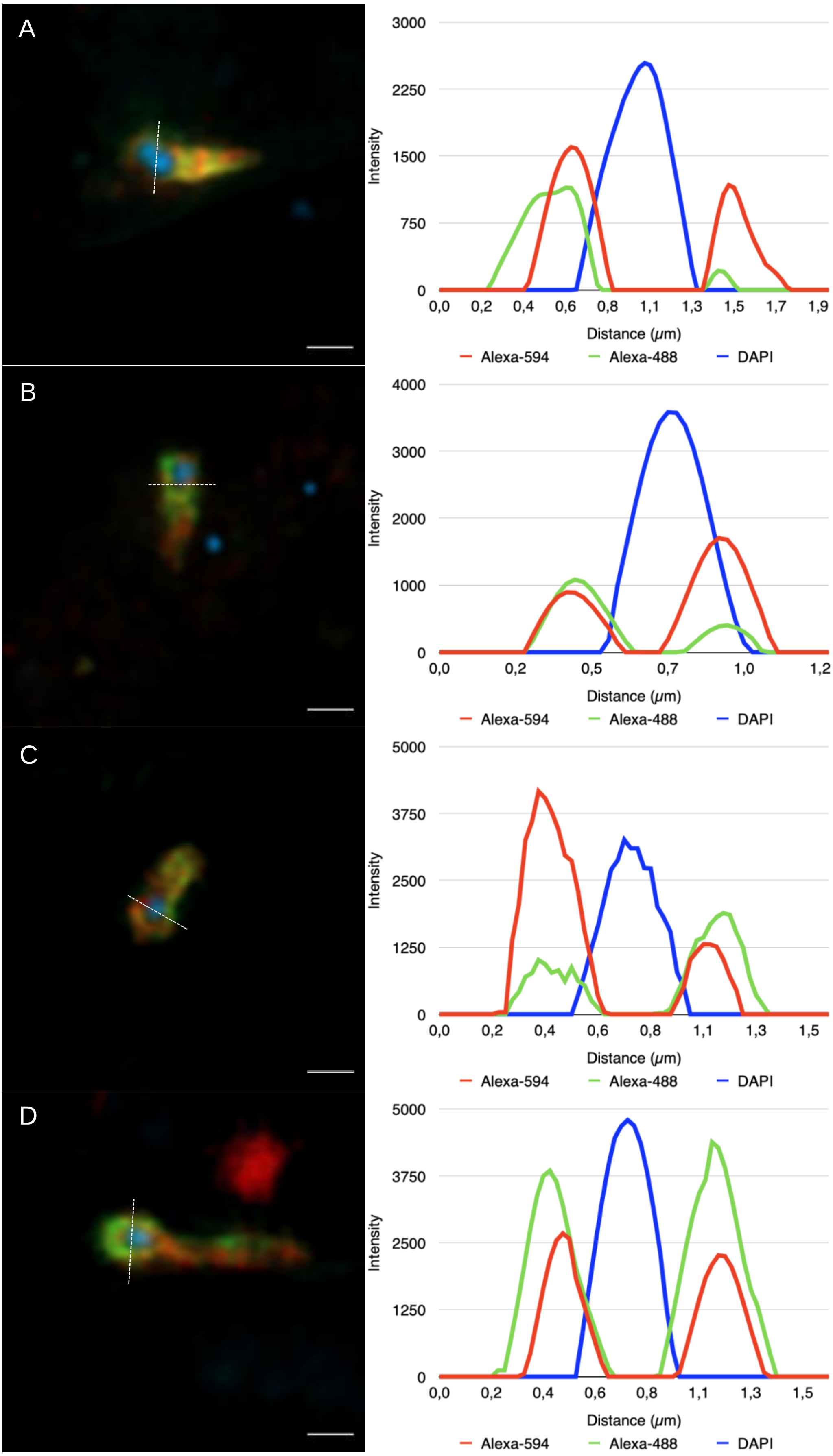
A
B
C
D
Intensity
Distance (µm)
Alexa-594
Alexa-488
DAPI

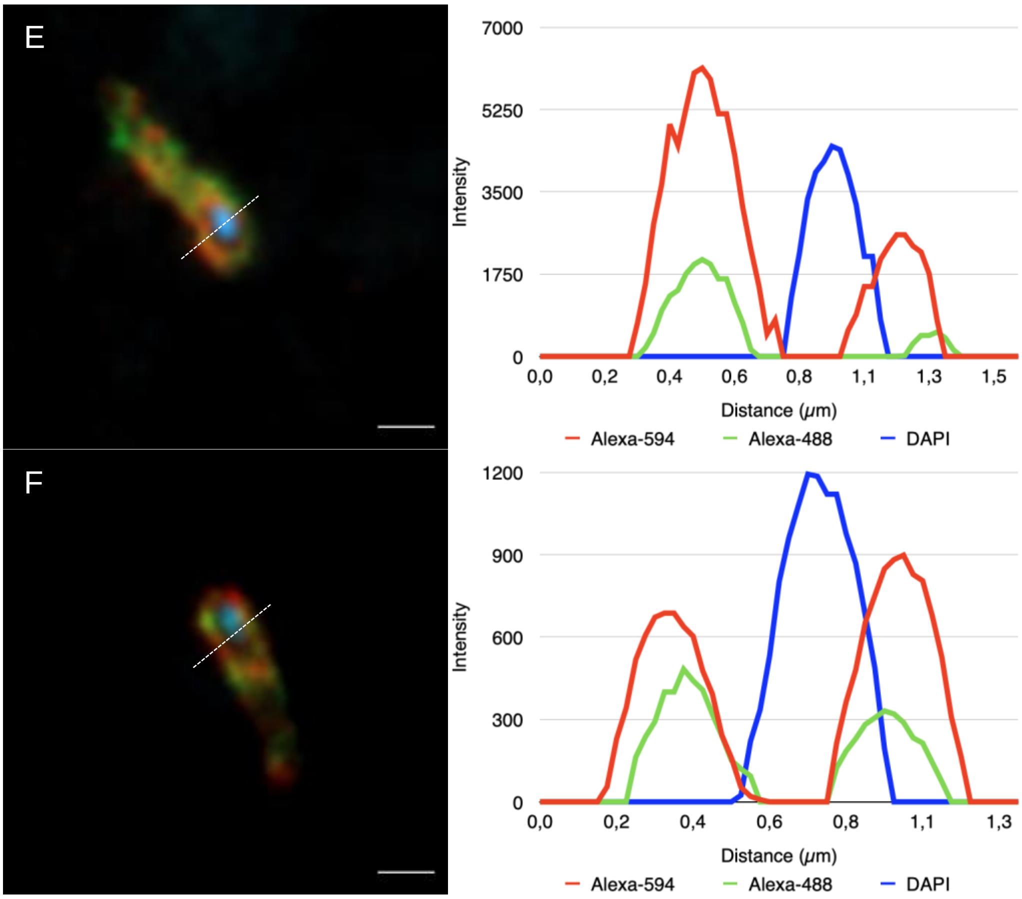

Supplementary Fig. 2 Line intensity profiles of DAPI and FISH signals in detected Hodarchaeales-related cells. Measurement was performed in ZEN Black software using the maximum intensity projection of detected cells acquired in super-resolution confocal laser scanning microscopy. Dashed lines indicate the position at which the fluorescence intensity line profiles were recorded. Background fluorescence is subtracted from the analysis. The scale bar is 1 μm.

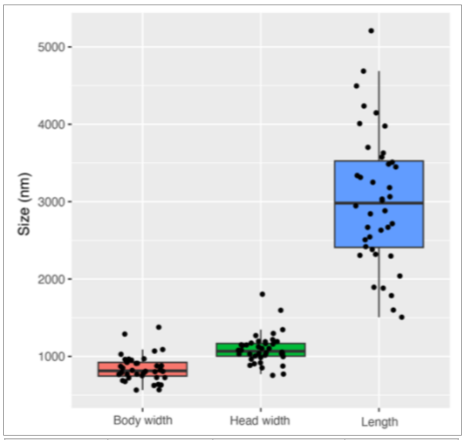

**Cell_1**	1377	1803	3629
**Cell_2**	1069	1160	3510
**Cell_3**	565	1037	1893
**Cell_4**	965	1190	2544
**Cell_5**	807	1048	2946
**Cell_6**	726	1081	2039
**Cell_7**	722	964	1786
**Cell_8**	1288	1596	4495
**Cell_9**	877	884	3701
**Cell_10**	921	1165	4009
**Cell_11**	910	1296	2418
**Cell_12**	825	1150	3485
**Cell_13**	630	852	2668
**Cell_14**	568	996	1882
**Cell_15**	624	1014	3314
**Cell_16**	772	1102	2715
**Cell_17**	687	1269	3064
**Cell_18**	766	1056	2668
**Cell_19**	806	1029	2382
**Cell_20**	1089	1143	2631
**Cell_21**	819	1191	4687
**Cell_22**	872	1038	4148
**Cell_23**	951	1005	3016
**Cell_24**	961	1078	3977
**Cell_25**	792	1030	1506
**Cell_26**	675	1102	2842
**Cell_27**	802	1030	3033
**Cell_28**	751	917	1599
**Cell_29**	850	1147	3449
**Cell_30**	1027	1196	3250
**Cell_31**	969	1345	2882
**Cell_32**	911	1217	3181
**Cell_33**	770	1124	2507
**Cell_34**	638	771	2319
**Cell_35**	823	904	3338
**Cell_36**	927	986	3575
**Cell_37**	879	1173	5209
**Cell_38**	727	755	2306
**Cell_39**	770	1035	4236
**Cell_40**	794	875	2297
Average	*843*	*1094*	*3028*
Median	*813*	*1067*	*2981*
St. Dev.	*171*	*194*	*864*
Minimum	*565*	*755*	*1506*
Maximum	*1377*	*1803*	*5209*

Supplementary Fig. 3 Measurement of sizes of Hodarchaeales-affiliated cells. The body and head widths correspond to that of the extended cell body and rounded expansion at one pole, respectively. Cell lengths and widths were measured using a line intensity profile drawn along the vertical and horizontal axes of the FISH signals. Imaging was performed by super-resolution confocal laser scanning microscopy. All values are reported in nm.

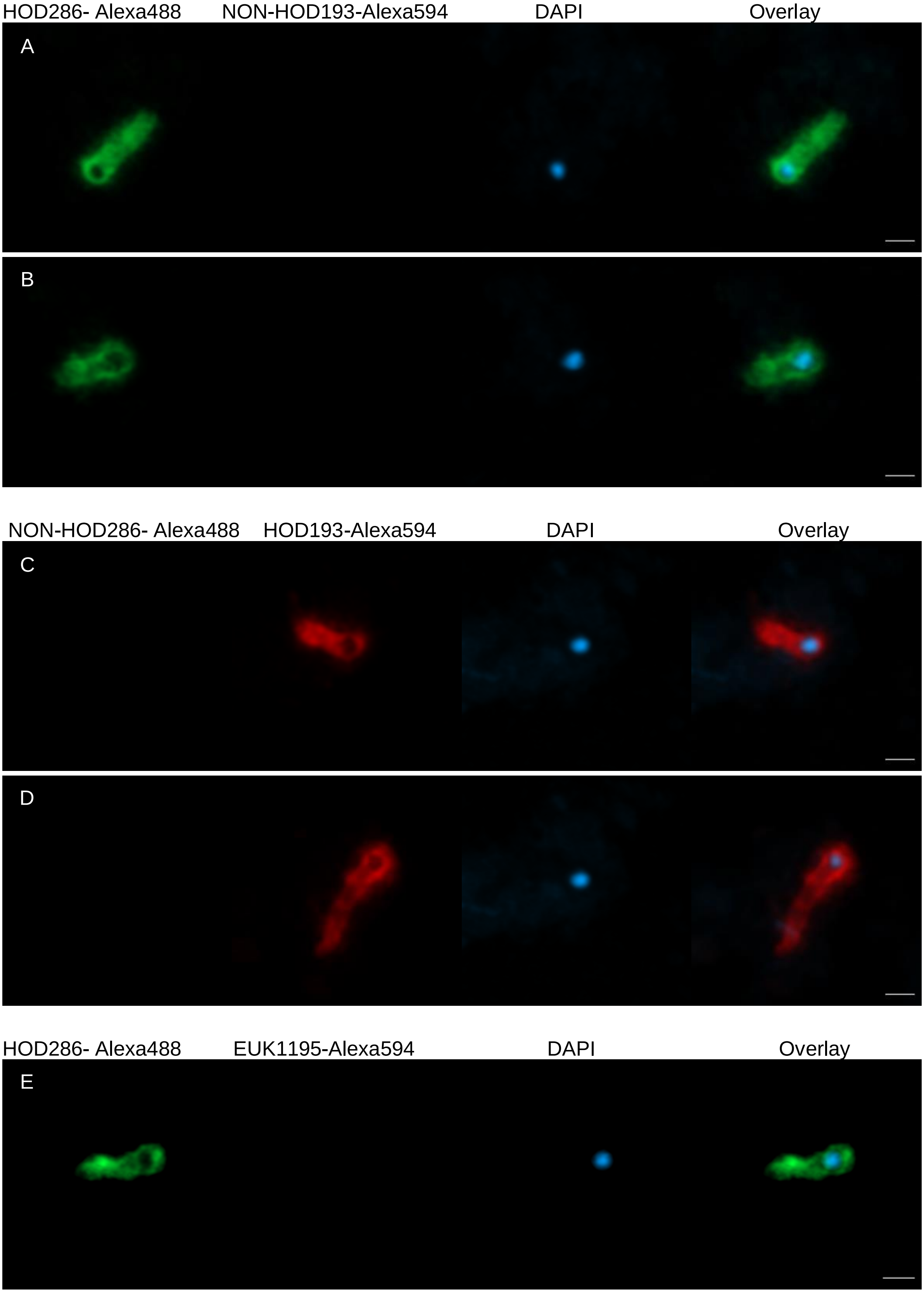
HOD286- Alexa488
NON-HOD193-Alexa594
DAPI
Overlay
A
B
NON-HOD286- Alexa488
HOD193-Alexa594
DAPI
Overlay
C
D
HOD286- Alexa488
EUK1195-Alexa594
DAPI
Overlay
E

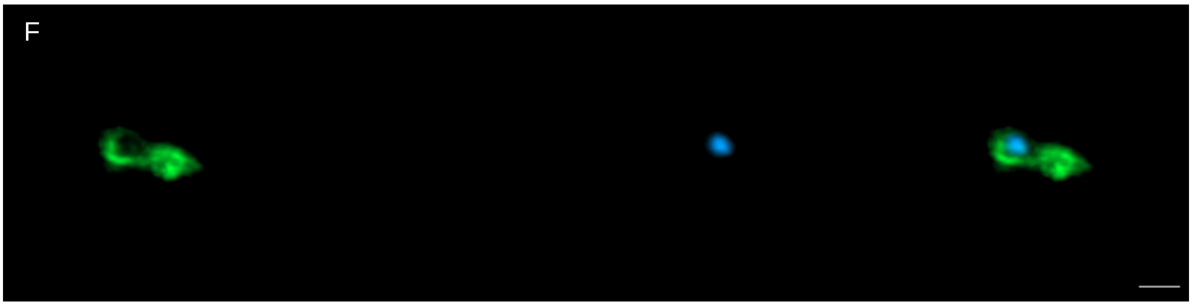

Supplementary Fig. 4 Control experiments for CARD-FISH hybridization of Hodarchaeales-related cells. A-D Nonsense probes NON-HOD193 and NOD-HOD286, which are reverse complementary to the corresponding probes, were used before/after hybridizations with HOD193 and HOD286 to confirm that detected morphotypes are not random probe binding or auto-fluorescent particles. E-F Negative control experiments with domain-specific eukaryote probe EUK1195 to verify the absence of any potential false-positive signals originating from non-target eukaryotic organisms. Maximum intensity projection of super-resolution confocal laser scanning microscope is depicted. Probe names and dyes for each panel are included. The scale bar is 1 μm.

Supplementary Table 1 Oligonucleotide probes used in this study. **A** Names, target groups, sequences, and formamide concentrations for newly designed Hodarchaeales probes together with non-sense and helper probes. **B** Number of the sequences in the target groups found in the NCBI core nucleotide database and the non-target hits at 0, 1, 2, 3 mismatches in the SILVA (v. 138) and NCBI core nucleotide databases.

A

Probe	Target	Probe sequence (5′ - 3′)	Formamide (%)	Reference
HOD193	Hodarchaeales	CCT AAG GCC CCG GAG GTT TGA ATA A	20	This study
HOD286	Hodarchaeales	GGC ACA CAC TCT CAT GTC CGG TAC	20	This study
NON-HOD193	Non-sense for HOD193	TTA TTC AAA CCT CCG GGG CCT TAG G	20	This study
NON-HOD286	Non-sense for HOD286	GTA CCG GAC ATG AGA GTG TGT GCC	20	This study
h1-HOD193	Helper for HOD193	ACT ACC ATA ATC CGC CGC AGA CCC AT	20	This study
h2-HOD193	Helper for HOD193	GAC ATT CCA GTA CAG CTT ATC TAT T	20	This study
h1-HOD286	Helper for HOD286	GCC CCT TGT CTC AGT GGC CAT CT	20	This study
h2-HOD286	Helper for HOD286	GGA TCA AAG GCT AGG TGG GCC GTT ACC C	20	This study
EUK1195	Eukaryotes	GGG CAT CAC AGA CCT G	35	Giovannoni et al., 1988

B

			Outgroup hits			
	Target group	Hits	0	1	2	3
HOD193	**20**	2	0	0	0	**3**
HOD286	**20**	11	0	0	0	5
